# A population-based approach to assessing diabetes management during COVID-19: insights from population data in Ontario, Canada.

**DOI:** 10.23889/ijpds.v7i3.2091

**Published:** 2022-08-25

**Authors:** Walter Wodchis, Yuqing Bai, Luke Mondor, Ruth Hall

**Affiliations:** 1 University of Toronto; 2 ICES

## Objectives

Diabetes management requires ongoing monitoring of diabetes care from primary care, specialist care and laboratory testing. COVID-19 led to changes in access to in-person care. The purpose of this research was to assess changes in the management of diabetes during the COVID-19 pandemic using population-linked datasets and population segmentation.

## Approach

We identified over 1.4 Million Ontarians with diabetes (approximately 10% of the population) with valid health insurance as of April 1, 2019 and April 1, 2020. We measured 11 indicators of diabetes management including laboratory testing for HbA1c and LDL (highlighted in this abstract). With screening indicators, we examined changes in the proportion of the population up-to-date with screening at the end of each fiscal year (March 31, 2020 and March 31, 2021) overall and according to population segments created using linked health data from primary care, home care, long term care and hospitals.

## Results

Overall screening rates that required laboratory testing for HbA1c and LDL fell substantially from 54% to 40% and 68% to 59% overall. Comparing across population segments, residents in Long Term Care facilities had the smallest changes in screening rates; individuals with low, medium and high complexity chronic conditions and end-of-life conditions had the largest changes; maternity, cancer, mental health and frail populations were in the middle. Differences according the primary care enrolment models (capitation vs fee-for-service) were relatively minor but patients who were not rostered to a primary care physician had the largest reductions in laboratory screening. Results for all 11 indicators will be shared in the presentation.

## Conclusion

COVID-19 was associated with substantial reductions in laboratory-based diabetes screening. Poor diabetes management is one of the strongest risk-factors for adverse outcomes in COVID-19. Rates of diabetes management declined most for at risk patient populations amplifying the need to differentially connect with patients to ensure ongoing care during the pandemic.

